# Colorectal Adenocarcinoma: A Comprehensive Narrative Review of Molecular Pathogenesis, Metabolic Vulnerabilities, and Therapeutic Potential of Sorghum Polyphenols

**DOI:** 10.1155/ancp/5527243

**Published:** 2026-06-01

**Authors:** Birhanemaskal Malkamu

**Affiliations:** ^1^ Department of Medical Laboratory Sciences, College of Health Sciences, Debre Tabor University, Debre Tabor, Ethiopia, dtu.edu.et

## Abstract

Colorectal cancer (CRC) remains a formidable global health challenge, characterized by uncontrolled cell proliferation and significant socioeconomic burden. Projections anticipate a substantial increase in new cases, straining healthcare systems worldwide. CRC, the most common histological subtype, originates from gland cells lining the colon and rectum, often preceded by benign polyps. Its development is complex, influenced by sporadic, familial, and inherited factors. Sporadic cases, accounting for 70%–80%, are linked to genetic mutations and epigenetic alterations, with lifestyle factors like physical inactivity, diet, obesity, smoking, and alcohol consumption playing significant roles. Familial cases (25%) suggest shared environmental influences, while inherited conditions (5%) like familial adenomatous polyposis (FAP) and Lynch syndrome involve germline mutation with high lifetime CRC risk. The molecular pathogenesis of CRC involves genomic instability, notably chromosomal instability (CIN), microsatellite instability (MSI), and the CpG island methylator phenotype (CIMP). Mitochondria, beyond ATP production, critically influence cellular processes. The mitochondrial metabolic theory (MMT) proposes impaired oxidative phosphorylation (OxPhos) drives genomic instability. While the Warburg effect is recognized, many tumors retain functional oxidative metabolism, often relying heavily on OxPhos. Key genetic mutations like *KRAS* and *BRAF* significantly impact CRC, influencing mitochondrial metabolism and tumor progression, and are associated with different prognostic outcomes. Emerging therapeutic strategies target metabolic vulnerabilities to overcome chemoresistance. Inhibition of mitochondrial genes and key enzymes, along with modulating calcium homeostasis, shows promise. Preclinical research highlights sorghum extracts as potential therapeutic agents. High‐phenolic sorghum bran extracts demonstrate anticancer action by suppressing proliferation, inducing apoptosis, and inhibiting migration and invasion. These effects involve targeting specific cancer pathways and influencing proteins like those in the Ras‐ERK pathway, β‐catenin, cMyc, cyclin D1, and surviving. Specific sorghum compounds like anthocyanidins, luteolin, and quercetin exhibit cytotoxicity against CRC cells, including those with *KRAS* and *BRAF* mutations. Further research is crucial to translate these preclinical findings into clinical applications, understand bioavailability, and account for individual differences in gut microbiota.

## 1. Background

Cancer stands as a major public health concern worldwide, marked by the uncontrolled and sustained proliferation of malignant cells [1]. Beyond hindering progress in how long people live, cancer also creates significant costs for society and the economy. These impacts vary depending on the specific type of cancer, where people live, and gender [2]. According to the latest International Agency for Research on Cancer (IARC) GLOBOCAN 2022 estimates, ~20 million new cases are diagnosed, and 9.7 million individuals succumbed to the disease [3, 4]. Projections indicate a significant surge in the cancer burden, with an anticipated 35 million new cases by 2050, primarily due to population growth. This escalating prevalence will further strain healthcare systems and impact communities worldwide [5, 6].

Among the diverse spectrum of malignancies, colorectal cancer (CRC) stands as a significant contributor to global mortality [7]. Worldwide, it ranks as the third most common cancer and is the second leading cause of cancer‐related deaths [8]. In 2022, ~1.9 million new cases of CRC were reported globally, accompanied by 900,000 deaths [9]. Future estimations suggest likely increment of the global CRC burden, with increases in both incidence and mortality. While mortality rates are currently increased in high‐income countries, developing countries are experiencing a rise in both the occurrences of CRC and associated fatalities. Interestingly, the number of new cases and deaths from CRC differs significantly in various parts of the world [10, 4, 11].

While previous literature has extensively documented the general chemopreventive properties of dietary polyphenols in CRC, there remains a significant gap in the systematic evaluation of sorghum‐specific polyphenols as metabolic modifiers. Sorghum (*Sorghum bicolor*) is uniquely characterized by high concentrations of 3‐doxyanthoyanidins, such as apigeninidin and luteolinidin, which possess greater chemical stability than anthocyanins found in other fruits and vegetables [12–14]. This review uniquely diverges from existing broader surveys by focusing specifically on the interplay between these unique sorghum metabolites and the “metabolic reprogramming” of CRC cells. By synthesizing recent evidence on how sorghum polyphenols disrupt glycolytic flux and mitochondrial bioenergetics, this review provides a novel framework for understanding sorghum not just as a fiber source but as a potent source of bioactive metabolic inhibitors in colorectal oncology.

## 2. Defining Colorectal Adenocarcinoma

Colorectal adenocarcinoma is defined as a malignant tumor originating from the gland cells that line the inside of the colon and rectum [15]. These gland cells produce mucus to lubricate and protect the colon and rectum. Adenocarcinoma represents the most prevalent histological subtype of CRC. The development of this malignancy is often preceded by the formation of benign growths known as polyps, particularly adenomatous polyps, which have the potential to undergo malignant transformation over time [16–18].

From an epidemiological perspective, CRC continues to be a significant health issue worldwide. In 2022, roughly 1.9 million new cases were diagnosed, and about 900,000 deaths occurred globally [19]. This positions CRC as the third most frequently diagnosed cancer globally. The development of CRC is a complex process influenced by a variety of factors, leading to the recognition of distinct molecular subtypes that exhibit different characteristics and responses to therapy [20].

## 3. Etiology of CRC

The etiology of CRC can be broadly categorized into sporadic, familial, and inherited forms [21, 22]. Understanding these different pathways is crucial for risk assessment, prevention strategies, and tailored treatment approaches.

The majority of CRC cases, accounting for ~70%–80%, are classified as sporadic [22–25]. These cases arise from a combination of genetic mutations and epigenetic alteration acquired over an individual’s lifetime, without a clear family history of the disease [26]. Several modifiable lifestyle determinants have been strongly correlated with an elevated risk of sporadic CRC.

Physical inactivity stands out as a preeminently scrutinized variable, with studies suggesting a potential for risk attenuation of ~25% contingent upon augmented physical activity levels [27, 28]. Other significant modifiable risk factors include dietary habits, particularly a long‐term diet high in red and processed meats and low in fruits and vegetables, as well as obesity, smoking, and excessive alcohol consumption [29]. Interestingly, there is an increasing incidence of early‐onset CRC (EO‐CRC), which appears to be primarily driven by these sporadic cases. This trend presents a challenge for early detection as these cancers occur before typical screening ages [30, 31]. While risk factors like family history, inflammatory bowel disease, obesity, alcohol, smoking, inactivity, and poor diet are linked to EO‐CRC, they do not fully explain the rising trend, leading some researchers to propose that EO‐CRC might represent a separate, more aggressive form of the disease [32, 33, 34].

Emerging evidence suggests that the metabolic landscape of EO‐CRC is distinct from late‐onset CRC (LO‐CRC), particularly regarding mitochondrial bioenergetics [31, 35]. While LO‐CRC typically reflects a cumulative attrition of mitochondrial function associated with cellular senescence and chronic oxidative stress, EO‐CRC often exhibits a more pronounced “metabolic plasticity.” Specifically, EO‐CRC cells demonstrate a heightened reliance on aerobic glycolysis and glutaminolysis [36], driven by distinct epigenetic modifications and mitochondrial DNA (mtDNA) stress signatures that differ from age‐related genomic decay [25]. This metabolic reprogramming in younger patients is frequently associated with an overactive PI3K/AKT/mTOR signaling axis, which enhances nutrient uptake to support rapid proliferation and chemoresistance [37]; consequently, the mitochondrial dysfunction in EO‐CRC appears less as a byproduct of aging and more as a proactive adaptation to exogenous metabolic stressors, necessitating age‐stratified approaches to metabolic targeting.

Approximately 25% of CRC cases exhibit a familial link, characterized by a higher incidence of CRC within a family compared to the general population, but without a clearly defined inherited syndrome [38–40]. This suggests a role for shared environmental factors, common lifestyle choices, or the combined effect of multiple low‐penetrance genetic variants that individually confer a modest increase in risk.

Hereditary conditions account for a smaller proportion, about 5%, of the total CRC burden [7, 40, 41]. These cases are characterized by germline mutations in specific genes that significantly increase an individual’s lifeline time risk of developing CRC. Two prominent inherited CRC syndromes include familial adenomatous polyposis (FAP) and Lynch syndrome [42]. FAP is an inherited disorder resulting from germline mutations in the *APC* gene located on chromosome 5q21 [43, 44].

FAP‐affected individuals are characterized by the early onset of numerous colorectal polyps, often in the teenage years, and face a near 100% lifetime risk of developing colorectal carcinoma, typically by the age of 40 if prophylactic colectomy is not performed [25, 45]. Certain variants of FAP, such as Gardner syndrome, may also involve extracolonic manifestations including osteomas and soft tissue tumors [46, 47].

Lynch syndrome, identified as hereditary nonpolyposis CRC (HNPCC), is caused by inherited defects in the DNA mismatch repair (dMMR) system, specifically in the MLH1, MSH2,MSH6, or PMS2 genes [48, 49]. This syndrome accounts for ~3% to 5% of all CRC diagnoses [50]. Individuals with Lynch syndrome have an increased risk not only for colorectal and endometrial cancers but also for a wider spectrum of other malignancies, including ovarian, stomach, small bowel, and urinary tract cancers [51]. CRC in Lynch syndrome patients often develops at a younger age, typically before the age of 50. Besides FAP and Lynch syndrome, other rarer inherited conditions such as Peutz–Jethers syndrome and MUTYH‐associated polyposis (MAP) also contribute to an increased risk for CRC [52].

## 4. Interplay of Lifestyle, Environmental Factors, and Disease Pathophysiology

Recent research trends emphasize that the global variation in CRC incidence is not merely a result of exposure but an interaction between environmental stressors and individual pathophysiology [53]. Lifestyle factors, including high‐red‐meat diets and sedentary behavior, influence disease mechanisms through epigenetic modifications and gut microbiome alterations [54, 55]. These environmental exposures can trigger chronic inflammation, which differentially affects molecular pathways such as the Wnt/β‐catenin signaling, explaining why individuals with similar lifestyles may experience vastly different clinical outcomes [56, 57]. Furthermore, the “long‐term” influence of these factors suggests a cumulative effect on the colonic epithelium, highlighting the necessity for personalized prevention strategies that account for an individual’s unique molecular landscape [58, 59].

### 4.1. Integrating Risk Factors and Pathophysiology: The Molecular Pathological Epidemiology (MPE) Framework

The global heterogeneity in CRC incidence and clinical outcomes cannot be fully elucidated by analyzing risk factors and tumor pathology in isolation. Instead, a MPE approach is required to bridge the gap between macrolevel environmental exposures and microlevel somatic alterations [60, 61]. MPE posits that each CRC case is unique, resulting from the complex interplay between the host’s exposome including diet, obesity, and gut microbiota and the molecular features of the malignancy.

Long‐term lifestyle behaviors do not merely increase cancer risk but actively shape the tumor microenvironment (TME) and molecular signatures [62, 63]. For instance, high‐fat western diets are strongly associated with the CpG island methylator phenotype (CIMP) and microsatellite instability (MSI), specifically via the modulation of the gut microbiome [63, 64]. These dietary patterns can induce chronic low‐grade inflammation, leading to epigenetic silencing of tumor suppressor genes [65, 66]. This explains why patients with similar clinical stages may experience differential disease progression; their tumors possess distinct molecular “fingerprints” carved by years of specific environmental influences [67].

Recent trends in MPE have expanded into immune‐epidemiology, investigating how lifestyle factors like physical activity can enhance T‐cell infiltration within the tumor [68, 69]. This provides a mechanistic link between behavioral interventions and improved survival rates. By adopting this integrated framework, we move beyond generic risk assessments toward a precision medicine model, recognizing that the “long‐term influence” of the environment dictates the individual molecular landscape of the tumor and its subsequent response to therapeutic agents.

### 4.2. The Prospective Cohort Incident‐Tumor Biobank Method (PCIBM)

To effectively implement the MPE framework, modern research has shifted toward the PCIBM [70]. Unlike traditional case–control studies that may suffer from recall bias, the PCIBM integrates longitudinal data on lifestyle habits and environmental exposures collected before cancer diagnosis with high‐dimensional molecular analysis of the resulting tumor tissue [71]. This methodology has been instrumental in identifying how long‐term exposures, such as dietary fiber intake or physical activity, specifically correlate with BRAF‐mutated or MSI‐high tumor subtypes [72]. By utilizing the PCIBM, researchers can establish a temporal link between chronic risk factors and subsequent molecular alterations, providing a roadmap for precision prevention and evidence‐based clinical practice. As a prototypical disease for this approach, CRC research utilizing PCIBM represents a promising frontier for discovering novel biomarkers that reflect the cumulative impact of a patient’s unique environment on tumor pathophysiology.

A distinctive strength of the PCIBM lies in its ability to capture the exposome, the totality of environmental exposures across an individual’s life course, and map it against the evolutionary trajectory of the tumor. By collecting biospecimens (such as blood or stool) at multiple time points years before the incident cancer occurs, researchers can observe the prediagnostic transition of the gut microbiome and systemic inflammatory markers [72, 73]. This longitudinal granularity allows for the identification of “molecular windows of susceptibility,” where specific dietary patterns or environmental toxins exert their maximal epigenetic influence on the colonic epithelium. Consequently, the PCIBM moves the field beyond static associations toward a dynamic understanding of how chronic stressors facilitate the “macro‐to‐micro” transition in carcinogenesis.

Recent advancements in PCIBM have integrated digital pathology and artificial intelligence to perform spatial transcriptomics on biobanked tissues [73]. This facilitates an “immuno‐MPE” approach, which investigates how long‐term habits, such as vitamin D intake or obesity, differentially shape the infiltration of T cells and myeloid‐derived suppressor cells within the TME [70, 74]. Such high‐dimensional data, derived from prospective cohorts, are pivotal for identifying high‐risk individuals who may benefit from personalized screening or targeted immunoprevention. By bridging the gap between population‐level lifestyle data and cell‐specific molecular markers, the PCIBM establishes a new paradigm for understanding the heterogeneity of CRC outcomes and refining clinical decision‐making.

## 5. Molecular Pathogenesis of CRC

The development of CRC is a multistep process driven by the accumulation of genetic and epigenetic alterations. A key feature of this process is genomic instability, which encompasses various mechanisms that lead to an increased rate of mutations and chromosomal abnormalities [24, 75]. In CRC, at least three distinct pathways of genomic instability have been identified: chromosomal instability (CIN), MSI, and the CIMP [76, 77].

CIN is the most prevalent form of genomic instability in CRC, observed in ~65%–70% of sporadic cases [78, 79]. It is characterized by an elevated rate of gains or losses of whole chromosomes or large chromosomal segments, resulting in aneuploidy (abnormal chromosome number) and a high frequency of loss of heterozygosity (LOH). Genes such as *APC*, *KRAS*, *and TP53* are frequently implicated in the CIN pathway [80, 81]. This widespread genetic instability facilitates tumor progression by disrupting the normal function of numerous genes involved in cell growth, survival, and differentiation.

MSI is another important pathway, occurring in about 15% of sporadic CRCs. MSI arises due to a deficiency in the dMMR system, often caused by epigenetic silencing of the *MLH1* gene promoter in sporadic cases. MSI‐high tumors are often located in the right colon, exhibit increased mucin production, and, in some stages, are associated with a better prognosis [82, 83]. The CIMP involves the hypermethylation of CpG islands in the promoter regions of various genes, leading to their transcriptional silencing [84]. CIMP is observed in a significant proportion of sporadic CRCs, estimated to be around 40%. Notably, there is a frequent overlap between CIMP‐high and MSI‐high tumors [85, 86]. CIMP‐high tumors are often associated with a proximal colon location, older age, and specific genetic alterations, such as *BRAF* mutations [18, 87]. These three mechanisms of genomic instability—CIN, MSI, AND CIMP—are not always mutually exclusive and can interact, contributing to the complex and heterogeneous metabolic landscape observed in different CRC subtypes [88, 89].

## 6. Metabolic Consequences of Genomic Instability Subtypes on Mitochondrial Function

The three principal genomic instability pathways in CRC—CIN, MSI, and the CIMP—are not merely genetic and epigenetic classifiers but also exert distinct influences on mitochondrial metabolism and bioenergetics [76, 90, 91].

CIN. CIN, present in ~70% of CRCs, is characterized by aneuploidy and LOH [92, 93]. Recent multiomics analyses integrating transcriptomic and proteomic data from patient‐derived organoids have uncovered significant associations between CIN and dysregulated mitochondrial metabolism [94]. Specifically, CIN‐high tumors exhibit altered expression of mitochondrial electron transport chain (ETC) components and increased reliance on glycolytic pathways, suggesting that the genomic burden imposed by CIN drives a metabolic shift away from oxidative phosphorylation (OxPhos) [95]. This association is further supported by the inverse relationship between CIN markers (such as 18q LOH) and CIMP, where CIN‐dominant tumors display global hypomethylation patterns that may indirectly affect nuclear‐encoded mitochondrial genes [96, 97].

MSI. MSI‐high tumors, arising from deficient dMMR, demonstrate direct mitochondrial consequences [98, 99]. The mitochondrial transcription factor A (TFAM), which is essential for mtDNA replication and maintenance, is frequently subject to truncating mutations in MSI CRC cells, affecting chemotherapy resistance [100, 101] . Consequently, MSI‐high tumors exhibit significantly reduced mtDNA copy number (mtDNA‐CN), and lower mtDNA‐CN correlates with worse disease‐free and overall survival in dMMR patients [102]. Furthermore, mtDNA methylation patterns, though technically challenging to detect, have been implicated in regulating tumor metabolism; genes associated with mtDNA methylation status in CRC include EPHB2, whose loss promotes a glycolytic phenotype, and FCN3, which affects immune surveillance [103, 104].

CIMP. CIMP involves promoter hypermethylation of tumor suppressor genes, leading to their transcriptional silencing [105]. While direct evidence linking CIMP to mitochondrial dysfunction is less established, CIMP‐high tumors frequently overlap with MSI‐high and BRAF‐mutant subtypes, indirectly inheriting the mitochondrial vulnerabilities described above [106, 107]. Moreover, CIMP‐associated hypermethylation of nuclear genes encoding mitochondrial proteins represents a plausible but underinvestigated mechanism. The inverse relationship between CIMP and CIN further suggests that CIMP‐positive tumors may rely more heavily on residual OxPhos capacity compared to CIN‐driven CRCs [108].

In summary, each genomic instability subtype creates a distinct mitochondrial metabolic landscape: CIN drives mitochondrial suppression and glycolytic adaptation, MSI directly reduces mtDNA‐CN and affects mitochondrial transcription, and CIMP may indirectly modulate mitochondrial gene expression through epigenetic silencing. These subtype‐specific metabolic vulnerabilities offer potential therapeutic opportunities for targeted intervention.

Mitochondria, beyond their well‐established role as cellular powerhouses for ATP production, are increasingly recognized for their pivotal involvement in various cellular processes, including synthesis, metabolism, signal transduction, and the regulation of cell death [109, 110]. The mitochondrial metabolic theory (MMT) posits that impaired OxPhos is a key driver of genomic instability and metabolic reprogramming in cancer [111, 112]. While the Warburg effect, characterized by increased glucose uptake and lactate production even under aerobic conditions, is a well‐known feature of cancer cells, recent findings challenge the traditional view that its primary role is to provide energy for rapid proliferation in CRC [113–115]. Instead, accumulating evidence suggests that the Warburg effect serves a dual, context‐dependent role in CRC: while it supports biosynthesis, a primary function is the reduction of reactive oxygen species (ROS) to mitigate oxidative stress and prevent apoptotic activation [116, 117]. This ROS‐centric role appears particularly relevant in CRC, where elevated mitochondrial ROS (mROS) due to genomic instability or mutant KRAS/BRAF signaling would otherwise trigger cell death [118]. However, this is context‐specific; in other malignancies such as aggressive lymphomas or hypoxic tumors, the Warburg effect prioritizes glycolytic ATP production to meet high energetic demands [119, 120, 115].

Current evidence indicated that many tumors, including CRC, retain functional oxidative metabolism, and many even heavily rely on OxPhos for ATP production. Even specific mitochondrial mutations in genes like SDH, IDH, and FH do not eliminate mitochondrial dependence; instead, cancer cells adapt mitochondrial function for ROS production and the synthesis of components needed for growth [121, 122]. Furthermore, a bidirectional relationship exists between mitochondrial metabolism and genomic instability, where mitochondrial alterations can influence the accumulation of DNA mutations, and conversely, DNA damage can impact mitochondrial function [123].

While the MMT offers a compelling framework for understanding cancer metabolism, several limitations and controversies warrant consideration. First, the claim that OxPhos is universally impaired in cancer has been challenged by studies demonstrating that many tumors retain functional OxPhos and rely on it for ATP production under physiological conditions [107, 124]. Second, the existence and functional significance of mtDNA methylation, a key pillar of some MMT‐based models, remains methodologically contested, with traditional bisulfite sequencing prone to false‐positive signals due to the unique superhelical structure of the mitochondrial genome [125, 126]. Third, the causal direction between mitochondrial dysfunction and genomic instability remains debated; while MMT posits that respiratory damage drives nuclear mutations, others argue that mitochondrial alterations may be secondary consequences rather than primary drivers of tumorigenesis [127, 128]. Finally, mitochondria‐targeting therapies have shown mixed results in clinical settings, raising questions about whether MMT‐informed strategies can translate into effective treatments across heterogeneous CRC subtypes. These limitations underscore the need for advanced technologies, such as nanopore sequencing, to directly validate mtDNA modifications and for quantitative models that capture the bidirectional crosstalk between mitochondrial and nuclear genomes in colorectal carcinogenesis.

## 7. Influence of Key Genetic Mutations

Mutations in the *KRAS* and *BRAF* genes are prevalent in CRC and can significantly impact patient outcomes. These genes are key components of signaling pathways that regulate cell proliferation and survival [18].

Mutations in the *KRAS* gene are found in a substantial proportion of CRC cases, ranging from 30%–50% of tumors. The *KRAS* protein, which functions as a *GTPase* enzyme, is critically involved in several fundamental cell activities such as growth, specialization, and staying alive [129]. Studies have shown that *KRAS* mutations can modulate mitochondrial metabolism in CRC. Specifically, *KRAS*‐mutated CRC exhibits lower ADP‐activated respiration compared to wild‐type tumors, yet still higher than normal tissue, suggesting an oxidative phenotype [130]. The impact of *KRAS* mutation on mtDNA‐CN is less consistent, with some studies reporting lower levels and others indicating higher levels [131, 132]. Beyond mitochondrial metabolism, *KRAS* mutations influence the TME, promote cancer stem cell formation, activate macropinocytosis (a nutrient scavenging pathway), and are involved in cell competition [133–135]. Given the challenges in directly targeting *KRAS*, current research focuses on understanding its downstream effector functions and the metabolic vulnerabilities it creates [136, 137].

Mutations in the *BRAF* gene, particularly the V600E mutation, are less frequent than *KRAS* mutations in CRC, occurring in ~8%–12% of cases [138, 139, 140]. These mutations are frequently associated with a minor prognosis. *BRAF*‐mutated CRC tends to exhibit reduced mitochondrial respiration and altered mitochondrial outer membrane permeability, indicating a glycolytic phenotype. Similar to *KRAS*, *BRAF* mutations have been linked to lower mtDNA‐CNs. The BRAF V600E contributing mutation is strongly associated with sporadic MSI‐H CRC and might be linked to a worse prognosis in metastatic MSI‐H CRC [139]. Mechanistically, BRAF V600E mutation leads to higher levels of mitochondrial fission factor DRP1, resulting in fragmented mitochondria and to the glycolytic phenotype observed in these tumors [141, 142]. The distinct mitochondrial and metabolic features of *KRAS*‐mutant versus BRAF‐mutant CRC are summarized in Table 1.

**Table 1:** Comparative mitochondrial, metabolic, and therapeutic features of KRAS‐mutant versus BRAF‐mutant colorectal cancer.

Feature	KRAS‐mutant CRC	BRAF‐mutant CRC
Mitochondrial		
Mitochondrial respiration	Lower ADP‐activated respiration vs. wild‐type; still higher than normal tissue (oxidative phenotype)	Reduced mitochondrial respiration; altered outer membrane permeability (glycolytic phenotype)
mtDNA copy number	Inconsistent (reported both lower and higher levels)	Generally lower mtDNA copy number
Mitochondrial morphology	Less characterized	Fragmented mitochondria via DRP1 upregulation
Metabolic		
Metabolic phenotype	Glutamine dependence; oxidative phenotype	Glycolytic preference; reduced OxPhos
Key metabolic pathways	Macropinocytosis (nutrient scavenging); cell competition	Warburg effect dominance
ROS handling	Adapts to oxidative stress	Higher baseline ROS; vulnerable to further oxidative challenge
Therapeutic vulnerabilities		
Direct targeting	Difficult (historically undruggable); recent KRAS G12C inhibitors emerging	BRAF V600E inhibitors available (vemurafenib and encorafenib)
Synthetic lethality	MEK inhibitor + autophagy inhibitor combinations	BRAF inhibitor + EGFR inhibitor combinations
Metabolic targeting	Glutaminase inhibitors; macropinocytosis inhibitors	Glycolysis inhibitors; DRP1‐targeted therapies
Prognosis	Variable; context‐dependent	Generally worse prognosis, especially in metastatic MSI‐high CRC

*Note:* Sources Gao and Zheng [142], Kerk et al. [134], Shi et al. [138], van Gisbergen et al. [132], and Žilinskas et al. [143].

Recent evidences advocates that phenolic extracts from sorghum own therapeutic potential against CRC, possibly persuading pathways involving *KRAS* and *BRAF* [144, 145]. These extracts have been shown to decrease the levels of several key proteins involved in cancer growth and survival signaling, including factors in the Ras‐ERK pathway, β‐catenin, cMyc, cyclin D1, and Survivin [146, 147]. This regulatory effect has been suggested to involve p53 in some experimental contexts, though it may also occur through p53‐independent mechanisms including suppression of the WNT/β‐ catenin signaling pathway, particularly when the APC gene is mutated [148, 149]. It remains unclear whether these effects are retained in p53‐mutant CRC models, which represent ~40%–50% of CRCs.

Limitation regarding p53‐mutant CRC models. It is important to note that p53 mutations are present in ~40%–50% of CRCs, particularly in late‐stage and chemoresistant disease [150–152]. The majority of studies cited herein, including those demonstrating p53 involvement, have primarily utilized CRC cell lines with wild‐type p53 or unspecified p53 status. Whether the anticancer effects of sorghum polyphenols, including apoptosis induction, cell cycle arrest, and signaling pathway modulation, are retained in p53‐mutant CRC models remains an open question. While structurally related polyphenols (e.g., curcumin, resveratrol, and quercetin) have demonstrated p53‐independent proapoptotic activity through mROS generation and AMPK activation [153, 154], direct evidence for sorghum‐specific extracts in p53‐mutant cells is currently lacking. Future studies using isogenic CRC cell lines with defined p53 status (wild‐type, null, and common mutants such as R175H, R248W, or R273H) are warranted to determine whether sorghum polyphenols retain efficacy in p53‐mutant contexts, which represent a large segment of the CRC patient population.

Sorghum extracts have demonstrated the ability to hinder cancer cell growth, induce necrosis in mouse models, decrease the expression of IGF‐1R (insulin‐like growth factor 1 receptor) and VEGF (involved in cell proliferation), and boost cell cycle inhibitors [155]. Specific compounds found in sorghum, such as the anthocyanins apigeninidin and luteolinidin from red sorghum, have been identified as inhibitors of cell growth in colon cancer cells. Furthermore, luteolin and quercetin, also present in sorghum, have shown promise as potent inhibitors of proliferation and inducers of apoptosis in both *KRAS*‐ and *BRAF*‐mutated human CRC cells [156]. Even if there is intensive research going on this area, there is a need for research in the role of phenolic compounds inside the metabolism of mitochondria and its participation or repair mechanism in the alignment of p53 and also outside of the mitochondria environment.

## 8. Role of Sorghum Polyphenols in Treatment of Chemoresistant CRC

Polyphenols, in general, exhibit several properties that could be beneficial in overcoming chemoresistance. This could be in modulation of signaling pathways: Many snippets highlight the ability of polyphenols to interfere with signaling pathways that are often deregulated in cancer and can contribute to drug resistance. For instance, polyphenols can affect the p13k/AKT/mTOR, MAPK/ERK, and Wnt/β‐catenin pathways. Dysregulation of these pathways is frequently implicated in the development of chemoresistance in CRC. By modulating these pathways, polyphenols might help to resensitize cancer cells to chemotherapy [157].

Noteworthy, the majority of cited sorghum studies utilized standard CRC cell lines (e.g., HCT‐116, HT‐29, and Caco‐2) rather than established chemoresistant models; therefore, the discussion of chemoresistance reversal remains largely theoretical at present.

### 8.1. Induction of Apoptosis

Several studies show that polyphenols can induce apoptosis in CRC cells. Chemoresistant cells often have defects in their apoptotic pathways. Polyphenols might be able to trigger apoptosis through alternative mechanisms, bypassing the resistance mechanisms [158].

### 8.2. Antioxidant and Anti‐Inflammatory Properties

Chronic inflammation and oxidative stress can contribute to cancer progression and chemoresistance. Polyphenols are known for their antioxidant and anti‐inflammatory activities. By reducing oxidative stress and inflammation, polyphenols might create a more favorable environment for chemotherapy to be effective [159–161].

### 8.3. Modulation of Drug Efflux Pumps

Some chemoresistance mechanisms involve the overexpression of efflux pumps that actively remove drugs from cancer cells. While not explicitly mentioned in the context of sorghum polyphenols in these snippets, other polyphenols have been shown to inhibit these pumps in various cancers, potentially increasing the intracellular concentration of chemotherapeutic agents [162, 163, 164].

A research discussing an extract of HDW plant extract of polyphenol suggests that it may overcome drug resistance of HCT‐8/5‐FU cells by downregulating the expression of ABC subfamily G member 2 (ABCG2) AND P‐ glycoprotein (P‐gp) or via inhibiting the phosphorylation of the p13k/akt signaling pathway [165, 166]. This highlights mechanisms by which natural compounds can potentially reverse chemoresistance, and sorghum polyphenols might act through similar pathways.

Of direct relevance, Ravisankar et al. [167] demonstrated that apigenin and quercetin, flavonoids present in sorghum, synergistically downregulated ABC transporter expression (3‐ to 40‐ fold) and inhibited P‐gp ATPase activity in Caco‐2 cells, suggesting direct binding and inhibition. However, direct evidence in chemoresistant CRC models remains limited and warrants further investigation.

Luteolin and quercetin, flavonoids found in sorghum, are mentioned as potent inhibitors of proliferation and inducers of apoptosis in both *KRAS*‐ and *BRAF*‐mutated human CRC cells [144, 168]. While not specific to chemoresistance, overcoming resistance is often a greater challenge in cancers with these mutations.

The antineoplastic efficacy of sorghum polyphenols in CRC is predicated on a multitargeted disruption of mitochondrial bioenergetics. Specifically, condensed tannins and 3‐deoxyanthocyanidins act as mitochondrial “metabolic stressors,” suggested to inhibit key enzymes in the tricarboxylic acid (TCA) cycle and the ETC [169]. This inhibition leads to a precipitous drop in ATP production and a concomitant increase in mROS [170, 171]. The resulting oxidative stress triggers the opening of the mitochondrial permeability transition pore (mPTP), facilitating the release of cytochrome c into the cytosol and committing the CRC cell to the intrinsic apoptotic pathway [153, 172, 173]. By forcing a shift away from efficient OxPhos, a state already compromised in the Warburg phenotype of colorectal cells—sorghum metabolites exploit the inherent mitochondrial vulnerabilities of the TME, effectively starving the cancer cells of the energetic precursors required for rapid proliferation.

**Figure 1:**
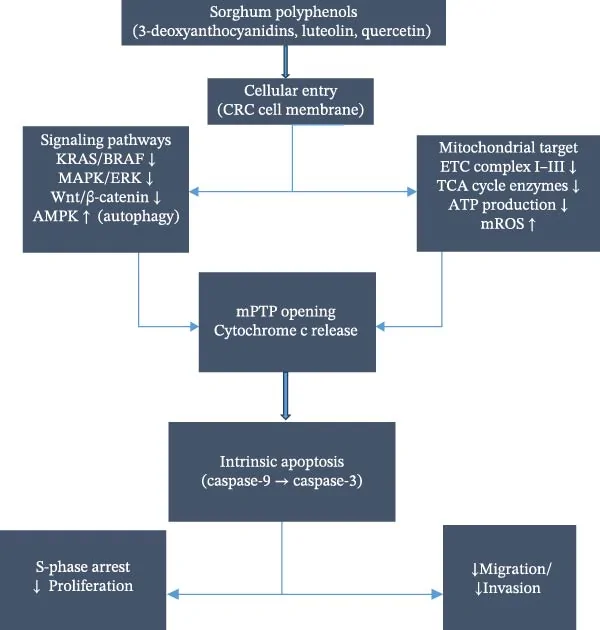
Unifying mechanistic model of sorghum polyphenol action on mitochondrial metabolism in colorectal adenocarcinoma.

As presented in Figure 1, sorghum‐derived polyphenols (3‐deoxyanthocyanidins, luteolin, and quercetin) enter CRC cells and exert dual effects: (1) proposed mitochondrial targeting (ETC complex I–III inhibition, TCA cycle suppression, ATP depletion, and mROS elevation) (though direct binding remains unconfirmed) leading to mPTP opening and cytochrome c release and (2) modulation of signaling pathways (KRAS/BRAF/MAPK/ERK suppression, Wnt/β‐catenin inhibition, and AMPK activation). These converge on intrinsic apoptosis (caspase‐9→caspase‐3), S‐phase arrest, reduced proliferation, and decreased migration/invasion. Concurrently, sorghum polyphenol treatment is associated with reduced activity of oncogenic signaling cascades including KRAS/BRAF/MAPK/ERK and Wnt/β‐catenin while activating AMPK‐mediated autophagy, further reinforcing the antiproliferative and proapoptotic effects.

### 8.4. Sorghum‐Derived Polyphenols Enter CRC Cells and Exert Dual Effects

(1) Proposed mitochondrial targeting (ETC/TCA suppression, ATP depletion, and mROS elevation) leading to mPTP opening and cytochrome c release and (2) modulation of signaling pathways (KRAS/BRAF/MAPK/ERK/Wnt/β‐catenin suppression and AMPK activation). These converge on intrinsic apoptosis, cell cycle arrest, and reduced proliferation/migration.

Note: Most evidence is indirect (cell‐based/animal studies using crude extracts; see Table 2 for classification). Direct target binding in CRC models has not been conclusively confirmed.

**Table 2:** Classification of mechanistic evidence for sorghum studies.

Citation	Extract/compound	Model system	Key assays	Evidence type	Direct binding confirmed?
Yang et al. [156]	3‐Deoxyanthocyanidins	Cell‐free + cell‐based	Phase II enzyme activity	Direct (suggestive)	No (no SPR/ITC)
Smolensky et al. [174]	Sorghum bran extract	CRC cell lines	ROS, flow cytometry, and Western blot	Indirect	No
	High‐phenolic sorghum bran	HCT‐116 cells	Western blot (ERK and JNK)	Indirect	No
Lee et al. [175]	Polyphenol sorghum bran	Apc Min/+ mice + cells	Tumor counts and Western blot	Indirect	No
Seo et al. [169]	Sorghum seed extracts	Cancer cell lines	Apoptosis gene PCR	Indirect	No
	Sorghum extract	SH‐SY5Y cells	Mitochondrial assays	Indirect	No
Erdoğan et al. [154]	Quercetin and luteolin	CRC cell lines	Western blot (p‐ERK and p‐Akt)	Indirect	No
Xu et al. [168]	Review	N/A	N/A	N/A	N/A
Collins et al. [144]	Review	N/A	N/A	N/A	N/A

Abbreviations: CRC, colorectal cancer; ITC, isothermal titration calorimetry; NQO, NAD(P)H:quinone oxidoreductase; p‐ERK, phosphorylated extracellular signal‐regulated kinase; ROS, reactive oxygen species; SPR, surface plasmon resonance.

Even if the above points mentioned are key in understanding the role of polyphenols in chemoresistant therapy of CRC researches, this area needs rigorous research and understand the cellular along with immunological effects of polyphenols in chemoresistant CRC. The following are research gaps that need to be well studied. The translation from preclinical studies to clinical trials has been poor for dietary phenolic against CRC. This could be due to factors like bioavailability and the amounts needed to exert effects in humans often exceeding common dietary doses. In addition, the specific cause–effect relationship between biological activity and ingested polyphenols, including their metabolites, is not fully understood, particularly in the context of drug‐resistant CRC. Finally, there is a need for more research on how individual differences in gut microbiota affect the metabolism and efficacy of phenolic compounds in drug resistance CRC prevention and treatment.

### 8.5. Role of Gut Microbiota

The gut microbiota critically influences polyphenol bioavailability and bioactivity. Sorghum polyphenols (e.g., 3‐deoxyanthocyanidins and tannins) undergo extensive colonic biotransformation via microbial enzymes, converting them into smaller phenolic acids and aglycones that may exhibit enhanced anticancer activity compared to their parent compounds [176, 177]. Conversely, microbial fermentation can also generate potentially toxic byproducts depending on individual microbiome composition, and the prebiotic‐like effects of polyphenols may modulate gut immune responses relevant to CRC [178]. To date, no studies have directly examined microbiota‐mediated metabolism of sorghum polyphenols in CRC models. Future research should integrate microbiome profiling with pharmacokinetic and efficacy studies to identify microbial signatures associated with favorable versus adverse responses.

### 8.6. Limitation of Poor Bioavailability

Sorghum polyphenols have limited systemic bioavailability. Alban and Manuel [179] reported flavone absorption at 30%–42% but only 1%–11% for 3‐deoxyanthocyanidins, consistent with achievable plasma concentrations of 0.1–5 µM. Al‐Mamary et al. [180] showed that high‐tannin sorghum inhibited digestive enzymes (α‐amylase by 77%) and reduced mineral absorption, raising toxicity concerns at higher doses. Yet most in vitro anticancer studies use 10–100 µM—10‐ to 100‐fold higher than physiologically relevant levels. Collins et al. [144] demonstrated that simulated digestion increased bioaccessibility 2‐fold, suggesting processing strategies may help. Future studies should (i) test physiologically relevant concentrations (≤5 µM), (ii) use active metabolites, (iii) explore processing/formulation strategies (e.g., fermentation and nanoencapsulation), and (iv) include pharmacokinetic assessments.

### 8.7. Pathways to Clinical Translation

Despite robust preclinical evidence, clinical translation of sorghum polyphenols remains limited. I propose three realistic trial designs: The first is chemoprevention in high‐risk populations: a phase II randomized controlled trial in individuals with FAP or Lynch syndrome, using endoscopic polyp burden as the primary endpoint. Sorghum bran (standardized to 500–1000 mg polyphenols/day) versus placebo for 12 months. Second, adjuvant therapy with standard chemotherapy: a phase I/II dose‐escalation trial combining sorghum polyphenols with FOLFOX or FOLFIRI in stage III/IV CRC patients. Endpoints: safety, tolerability, and preliminary efficacy (response rate and PFS). Third, combination with immunotherapy: a phase II trial in MSI‐H CRC patients receiving anti‐PD‐1 therapy (e.g., pembrolizumab) with or without sorghum polyphenols. Endpoints: objective response rate and immune correlates (e.g., CD8+ T‐cell infiltration). Key challenges to address include bioavailability enhancement, standardized extract production, and identification of predictive biomarkers (e.g., p53 or MSI status).

### 8.8. Direct Evidence for Immune Modulation

Emerging studies demonstrate that sorghum polyphenols directly modulate immune responses. Cox et al. [181] demonstrated that sorghum polyphenol extract reduced IL‐6 and IL‐10 production, attenuated STAT3 nuclear translocation, and induced autophagy (LC3‐II) in RAW 264.7 macrophages. Rhodes and Kresovich [182] showed that 3‐deoxyanthocyanidin–rich accessions significantly decreased LPS‐induced TNF‐α and IL‐6, while proanthocyanidin‐rich accessions increased cytokine production, indicating genotype‐specific effects. Francis et al. [183] further reported that black sorghum extracts upregulated HO‐1 and eNOS, downregulated NOX4, and reduced MCP‐1 and ICAM‐1 expression in human endothelial cells under oxidative stress. Collectively, these findings confirm that sorghum polyphenols can modulate immune cell cytokine production, STAT/NF‐κB signaling, and oxidative stress responses. However, direct evidence in CRC immune microenvironments—including effects on T‐cell metabolism, PD‐L1 expression, and macrophage polarization—remains unexplored and warrants future investigation.

### 8.9. Limitation Regarding Mitochondrial Localization

While the studies cited demonstrate indirect evidence of mitochondrial engagement (e.g., reduced ATP, elevated mROS, and mPTP opening), direct evidence for mitochondrial localization or binding of sorghum polyphenols remains limited. Future studies should employ fluorescently labeled polyphenols (e.g., FITC‐conjugated 3‐deoxyanthocyanidins) for live‐cell colocalization with MitoTracker, mitochondrial fractionation coupled with LC‐MS/MS to quantify compound accumulation, and surface plasmon resonance or isothermal titration calorimetry to assess direct binding to ETC complexes. Additionally, use of mitochondrial‐targeted antioxidants (e.g., MitoTEMPO) could confirm whether mROS elevation is a primary mediator or a secondary consequence. Clinical trials testing sorghum polyphenols as chemoprevention, adjuvant therapy, or combination with immunotherapy are urgently needed, along with biomarker development to identify responsive patient subsets.

Furthermore, the efficacy of sorghum polyphenols in p53‐mutant CRC models, which represent ~40%–50% of CRC patients, has not been directly evaluated. Future studies using isogenic cell lines with defined p53 status are needed to determine whether the observed anticancer effects are retained in p53‐mutant contexts.

## 9. Diagnosis and Prognosis of CRC

The diagnosis and prognosis of CRC rely heavily on the TNM staging system and increasingly on the identification of novel pathological and molecular factors [184].

The TNM system for classifying CRC is a widely used standard that relies on evaluating three key aspects: the extent of the primary tumor (T), the involvement of regional lymph nodes (N), and the presence of distant metastasis (M). Each of these categories is further subdivided to provide a detailed anatomical description of the cancer’s spread. The overall stage, ranging from 0 to IV, is determined by combining the T, N, and M classifications with higher stages indicating more advanced disease. The following table summarizes the TNM staging of CRC [185, 186]. The TNM staging system, summarized in Table 3, provides a standardized framework for anatomical classification of disease extent.

**Table 3:** TNM staging of colorectal cancer [187].

Stage	T (tumor)	N (nodes)	M (metastasis)
0	Tis (carcinoma in situ)	N0 (no lymph node involvement)	M0 (no distant metastasis)
I	T1 or T2 (invades submucosa or muscularis propria)	N0	M0
IIA	T3 (invades subserosa)	N0	M0
IIB	T4a (invades visceral peritoneum)	N0	M0
IIC	T4b (invades adjacent organs/structures)	N0	M0
IIIA	T1–T2	N1 (1–3 regional lymph nodes)	M0
	T1	N1c (tumor deposits in subserosa/mesentery)	M0
IIIB	T3–T4a	N1	M0
	T1–T2	N2a (4–6 regional lymph nodes)	M0
IIIC	T3–T4a	N2a	M0
	Any T	N2b (7+ regional lymph nodes)	M0
IVA	Any T	Any N	M1a (metastasis to one distant site/organ)
IVB	Any T	Any N	M1b (metastasis to > one distant site/organ)
IVC	Any T	Any N	M1c (metastasis to peritoneal surface)

While the TNM system provides a valuable framework for staging, it primarily relies on quantitative measures and captures only a single snapshot of the tumor at a specific point in time [188]. This limitation has led to the investigation of novel pathological features that may offer additional prognostic information. Recent evidence indicates that the lymph node ratio (LNR), defined as the ratio of metastatic lymph nodes to the total number of examined lymph nodes, and the log odds of positive lymph nodes (LODDS), a statistical measure incorporating the number of positive and negative lymph nodes, outperform conventional nodal staging in predicting patient outcomes [189, 190]. These factors suggest their potential utility in refining current classifications to better stratify patients with similar TNM stages. Furthermore, tumor deposits (TDs), currently classified within the N1c category in the absence of lymph node metastasis, have been shown to have an independent impact on survival, with research advocating for a quantitative rather than dichotomous assessment [191]. While a quantitative assessment of tumor extension is important, it alone is insufficient to provide the full range of diagnostic, analytical, and potentially predictive information necessary for optimal treatment planning [192]. Therefore, a qualitative evaluation of the tumor’s characteristics, documenting its aggressiveness and providing insights into strategies for preventing its progression, is also crucial; typically, chemotherapy is administered when the tumor has spread to regional lymph nodes or when other adverse prognostic factors are present.

## 10. Therapeutic Strategies Targeting Metabolic Vulnerabilities in CRC

Targeting metabolic vulnerabilities has emerged as a promising strategy to overcome chemoresistance in CRC. Given the altered metabolic landscape of cancer cells, disrupting these pathways can selectively target tumor cells and enhance the efficacy of conventional therapies.

Inhibition of mitochondrial genes similar to *POLG* and *PINK1* can induce synthetic lethality in MLH1‐deficient cells [193]. This suggests a specific vulnerability in mismatch repair (MMR)‐deficient CRC that can exploit therapeutically. Werner helicase (WRN) has also been identified as a synthetic‐lethal target in mismatch repair deficient CRC, with its loss in MSI CRC inducing p53/PUMA‐mediated apoptosis [194]. Of note, this mechanism is specific to WRN inhibition and does not directly inform on sorghum polyphenol activity in p53‐mutant CRC.

Inhibitors targeting mitochondrial complex I, such as metformin and piericidin, have shown potential as antitumor treatments, and metformin can alleviate chemoresistance in CRC cells [195]. Curcumin, an NCLX inhibitor, exhibits antitumoral activity in MSI CRC cells by inducing mitochondrial calcium overload and ROS production [196]. Targeting ALDH1B1, a mitochondrial enzyme, has also been proposed as a therapeutic strategy, particularly for targeting cancer stem cells in CRC [197]. Furthermore, combining immunotherapy with drugs targeting FAM‐associated genes can achieve a synergistic antitumor effect in CRC [198].

## 11. Potential of Sorghum Extracts in CRC Therapy: Preclinical Evidence and Mechanisms of Action

Preclinical research has highlighted the potential of sorghum extracts a therapeutic agent for CRC. Researches have demonstrated that high‐phenolic sorghum bran extracts exhibit anticancer‐associated activities in human colon cancer cells by significantly suppressing cell proliferation in a dose‐dependent way. These extracts induce apoptosis and S‐phase growth arrest while also inhibiting cell migration and invasion. These effects are accompanied by altered expression of regulating apoptosis, cell cycle, and metastasis [174]. Furthermore, high‐phenolic sorghum bran extracts have been shown to stimulate DNA damage in association with the induction of ERK and JNK signaling pathways [175]. In vivo studies using a genetic colon cancer rodent model have shown that feeding with high‐phenolic sorghum bran for 6 weeks significantly suppressed tumor formation.

The mechanisms underlying the anticancer effects involve targeting specific cancer pathways that control cell growth and the spread of the tumor. Sorghum phenolic extracts have been shown to block TNF‐α–stimulated NF‐κB transactivation and IGF‐1–stimulated P13k/AKT pathway via the downregulation of β‐catenin transactivation. Furthermore, these extracts can trigger AMPK and autophagy, cellular processes that play a role in tumor suppression. Specific phenolic compounds that originate in sorghum, including 3‐deoxyanthocyanidin, vanillic acid, ferulic acid, and protocatechuic acid, exhibit cancer cell cytotoxicity [199]. Notably, luteolin and quercetin, flavonol present in sorghum, have been identified as potent inhibitors of proliferation and inducers of apoptosis in both *KRAS*‐ and *BRAF*‐mutated human CRC cells [154]. These compounds have been associated with decrease ERK phosphorylation in *KRAS*‐mutated cells and Akt phosphorylation in *BRAF*‐mutated cells, suggesting an impact on key signaling pathways involved in CRC progression.

### 11.1. Mechanistic Nature of Sorghum‐Induced Pathway Inhibition

It is essential to distinguish between the direct and indirect mechanisms of action for the sorghum polyphenols discussed herein. The majority of cited studies utilize crude or partially purified extracts in cell‐based or animal models. In these systems, the observed effects, such as decreased phosphorylation of ERK and Akt, or altered expression of apoptotic genes, represent indirect signaling modulation or gene expression reprogramming. While 3‐deoxyanthocyanidins are proposed to bind to active sites of cellular proteins responsible for tumor inhibition [171], definitive evidence of direct target binding (e.g., via biophysical assays such as SPR or cocrystallization) is currently lacking for most sorghum metabolites. Therefore, while the functional evidence for pathway suppression is robust, the precise primary molecular targets remain to be fully elucidated. Future investigations utilizing bioactivity‐guided fractionation and direct binding assays are required to confirm true ligand‐target engagement.

From a MPE perspective, the therapeutic efficacy of sorghum‐derived phenolics may be significantly modulated by the tumor’s underlying molecular signature. Recent evidence suggests that high‐phenolic extracts, specifically those rich in 3‐deoxyanthocyanidins, exhibit differential cytotoxicity based on genomic instability pathways; for instance, certain polyphenols show enhanced proapoptotic activity in microsatellite unstable (MSI‐high) cells compared to microsatellite stable (MSS) lineages by exploiting existing dMMR deficiencies [200, 201]. This subtype‐specific response suggests that sorghum polyphenols do not act as generic antiproliferative agents but rather interact with the unique “molecular fingerprints” of the tumor, such as CIMP status or BRAF mutations, which are themselves shaped by the patient’s long‐term environmental exposures [171, 202]. Incorporating such MPE‐informed insights into preclinical sorghum research is vital for transitioning toward precision nutritional interventions that account for interindividual tumor heterogeneity.

## 12. Conclusion

CRC remains a significant global health burden, necessitating continued research into its complex etiology, molecular pathogenesis, and therapeutic interventions. The understanding of CRC has evolved to recognize its heterogeneity, driven by diverse genomic instability pathways and key genetic mutations such as *KRAS* and *BRAF*. These molecular features not only influence disease progression but also impact responses to therapy, underscoring the importance of personalized medicine approaches.

The emerging field of targeting metabolic vulnerabilities, particularly mitochondrial dysfunction, offers promising strategies to overcome chemoresistance, a major challenge in CRC treatment. Inhibiting specific mitochondrial genes, targeting key metabolic enzymes, and modulating calcium homeostasis within mitochondria are areas of active investigation. Furthermore, the preclinical evidence supporting the anticancer potential of natural compounds like those found in sorghum extracts warrants further exploration. These extracts and their constituent phenolic compounds exhibit a range of antitumor activities and show efficacy against *KRAS* and *BRAF*‐mutated CRC cells, highlighting their potential as therapeutic or preventative agents.

Based on the findings presented, we prioritize three specific research gaps for future investigation: (i) mitochondrial calcium signaling: Whether sorghum polyphenols modulate mitochondrial calcium uptake (via MCU or NCLX) to trigger mPTP opening and apoptosis remains unknown and warrants direct investigation. (ii) p53‐mitochondria axis: Given that p53 regulates mitochondrial respiration and apoptosis, future studies should evaluate whether sorghum polyphenol efficacy is retained in p53‐mutant CRC models (~40%–50% of patients) using isogenic cell lines. (iii) Microbiome interactions: How gut microbiota metabolize sorghum polyphenols into active or toxic byproducts and how interindividual microbiome variation influences efficacy requires integrated microbiome‐metabolite studies.

Addressing these gaps will clarify the mechanisms of action, identify responsive patient subsets, and accelerate clinical translation of sorghum polyphenols in CRC.

## Author Contributions

Conceptualization and design of the study, acquisition of data, analysis and interpretation of data, investigation, methodology, project administration, supervision, validation, drafting the article, writing – reviewing and editing, final approval: Birhanemaskal Malkamu.

## Funding

The author received no specific funding for this work.

## Conflicts of Interest

The author declares no conflicts of interest.

## Data Availability

Datasets used and/or analyzed during this study are available from the corresponding author upon reasonable request.
